# Efficacy, effectiveness, and safety of rho-kinase inhibitors in uveitic glaucoma and ocular hypertension secondary to uveitis: a systematic review and meta-analysis

**DOI:** 10.1007/s00417-025-07111-1

**Published:** 2026-02-03

**Authors:** Blanca Aguilar-Barrera, Germán Mejía-Salgado, Juanita Cardona-López, Alejandra Reyes-Troncoso, Santiago David Acosta-Alzamora, David Santiago Escobar-Sánchez, Fábian Andrés Guauque, Ana María Camacho-Romero, Valentina Suarez-Carreño, Stefania García-García, Julian Beltrán-Briceño, Sofía Gómez-Vargas, Laura Zarate-Pinzón, Fernando Gómez-Goyeneche, Alejandra de-la-Torre

**Affiliations:** 1 Semillero de Investigación Semineuros, Centro de Neurociencia NeuroVitae, Instituto de Medicina Traslacional (IMT), Escuela de Medicina y Ciencias de la Salud, Universidad del Rosario, Bogotá, Colombia; 2 Ophthalmology Interest Group Universidad del Rosario (OIG-UR), Escuela de Medicina y Ciencias de la Salud, Universidad del Rosario, Bogotá, Colombia; 3 Neuroscience (NEUROS) Research Group, Neurovitae Research Center, Institute of Translational Medicine (IMT), Escuela de Medicina y Ciencias de la Salud, Universidad del Rosario, Carrera 24 # 63 C 69, Bogotá, Colombia; 4 Diagnóstico Ocular del Country, Bogotá, Colombia; 5 Health Sciences Faculty, UniversidadAutóónoma de Bucaramanga (UNAB), Bucaramanga, Colombia

**Keywords:** Uveitic glaucoma, Ocular hypertension, Rho-kinase inhibitors, Ripasudil, Netarsudil, Intraocular pressure

## Abstract

**Purpose:**

To evaluate the efficacy, effectiveness, and safety of Rho-kinase (ROCK) inhibitors in the management of uveitic glaucoma (UG) and ocular hypertension secondary to uveitis (OHT-SU) through a systematic review and meta-analysis.

**Methods:**

We systematically searched PubMed, EMBASE, Virtual Health Library, and medRxiv databases until October 29, 2024, for studies evaluating UG or OHT-SU patients treated with ROCK inhibitors. Eligible designs included case series with over 10 patients, cross-sectional studies, cohort studies, and randomized controlled trials. Risk of bias was assessed using the CLARITY tools and validated metrics such as those developed by Hoy et al., Hassan Murad et al., and the RoB-2 tool. A pressure (IOP) reduction meta-analysis at different follow-up points was conducted using R software. The study was registered in PROSPERO with the CRD42024618812 number.

**Results:**

Eleven studies (383 participants, 256 eyes) were analyzed. Some studies reported outcomes per eye, whereas others reported outcomes only per patient; thus, the number of eyes reflects only explicitly reported data. Based on the risk of bias assessment, two studies had a low risk of bias, four raised some concerns, and five were at high risk of bias. Pooled mean age was 64.2 ± 15.2 years (from 10 studies reporting age); sex distribution (50.5% male, 49.5% female, from 7 studies reporting gender).

ROCK inhibitors achieved a statistically significant reduction in IOP at 3 months (mean difference: -9.32 mmHg, 95% CI: -17.02 to -1.62). These effects were sustained at 12 months (-7.92 mmHg, 95% CI: -10.28 to -5.56). Combination therapy enhanced pressure control.

The most common adverse event was conjunctival hyperemia, with a pooled prevalence of 6% (95% CI: 3%-15%), followed by ocular pain and blurred vision. Importantly, no major safety differences were found between ripasudil and netarsudil. Additionally, ROCK inhibitors also improved surgical outcomes in UG patients.

**Conclusion:**

ROCK inhibitors demonstrate significant and sustained IOP-lowering effects in UG and OHT-SU with a favorable safety profile. Their additive effect with conventional treatments and potential benefits in surgical outcomes highlight their role as promising therapeutic agents. Further clinical trials are needed to compare different ROCK inhibitors and assess their long-term safety and effectiveness in different populations.

**Supplementary Information:**

The online version contains supplementary material available at 10.1007/s00417-025-07111-1.

## Key messages

***What is known*:**

- Rho-kinase (ROCK) inhibitors are a therapeutic option for lowering intraocular pressure (IOP) in glaucoma; however, their role in uveitic glaucoma (UG) and ocular hypertension secondary to uveitis (OHT-SU) remains poorly established.

***What is new*:**

- ROCK inhibitors significantly reduce IOP in UG and OHT-SU, with effects sustained up to 12 months.
- They have a favorable safety profile, with conjunctival hyperemia being the most frequent but mild adverse event, and no major differences between ripasudil and netarsudil.
- ROCK inhibitors not only enhance IOP control when combined with conventional therapies but may also improve surgical outcomes in UG patients.

**Supplementary Information:**

The online version contains supplementary material available at 10.1007/s00417-025-07111-1.

## Introduction

Uveitic glaucoma (UG) and ocular hypertension secondary to uveitis (OHT-SU) are serious complications of intraocular inflammation, affecting up to 20% of patients with uveitis [1, 2]. Although the pathophysiology is not yet fully understood, several mechanisms have been described to explain the development of glaucoma in patients with uveitis. These include increased protein levels in aqueous humor, leading to reduced trabecular meshwork (TM) permeability and obstruction of drainage pathways, inflammation-induced TM damage, steroid-induced ocular hypertension, and pupillary block caused by posterior synechiae formation, among others, highlighting the multifactorial nature of these complications [3].

The treatment of UG focuses on controlling intraocular inflammation and intraocular pressure (IOP) through anti-inflammatory and hypotensive medications. Corticosteroids, administered topically, periocularly, or systemically, remain the cornerstone of inflammation management but may generate steroid-induced ocular hypertension [4], especially in children, elderly individuals, and those with a history of primary open-angle glaucoma [5, 6]. For IOP control, beta-blockers, prostaglandin analogs (PGAs), alpha-adrenergic agonists (α2-agonists), and carbonic anhydrase inhibitors are commonly used [3]. However, the use of PGAs remains controversial due to their potential to exacerbate herpes simplex keratouveitis and cystoid macular edema [7], although some studies support their safety in UG [8]. Notably, beta-blockers and carbonic anhydrase inhibitors may be associated with an increased risk of macular edema [9]. Surgical intervention is often needed, but inflammation's high failure rates and postoperative complications limit its success [10]. These challenges underscore the guarded prognosis of UG and the urgent need for safer and more effective therapies.

Recently, Rho-kinase (ROCK) inhibitors have emerged as promising therapeutic agents due to their novel mechanism of action and favorable safety profile. Unlike conventional IOP-lowering therapies, which primarily reduce aqueous humor production (beta-blockers, carbonic anhydrase inhibitors, alpha-adrenergic agonists) or enhance uveoscleral outflow (PGAs), ROCK inhibitors act directly on the TM to relax its cells, reduce outflow resistance, and facilitate aqueous humor drainage through the Schlemm’s canal, which is responsible for the majority of aqueous humor drainage [11, 12]. Additionally, ROCK inhibitors offer neuroprotective and antifibrotic properties [11]. Current ROCK inhibitors include ripasudil, netarsudil, and fasudil. Clinical studies, particularly for ripasudil and netarsudil, have demonstrated significant reductions in IOP and a favorable safety profile in patients with UG and OHT-SU. The most commonly reported side effect was conjunctival hyperemia, with other adverse events being rare and generally mild [13–15].

Clinical trials have demonstrated the efficacy and safety of ROCK inhibitors, and real-world studies have shown promising outcomes across patient populations and less controlled settings [14, 15]. These data suggest that ROCK inhibitors can effectively lower IOP without worsening intraocular inflammation.

However, evidence remains fragmented, and comparative data with standard therapies are limited. A comprehensive synthesis of clinical trials and real-world studies is needed to clarify their long-term safety and therapeutic role. This systematic review and meta-analysis aimed to evaluate the efficacy, effectiveness, and safety of ROCK inhibitors in managing UG and OHT-SU, providing an integrated understanding of their value as emerging treatment options.

## Methods

### Type and design of the study

This systematic review and meta-analysis were conducted by the Preferred Reporting Items for Systematic Reviews and Meta-Analyses (PRISMA) guidelines (Supplementary Material 1) and were registered in the “International Prospective Register of Systematic Reviews” (CRD42024618812).

### Search methods for identifying studies

We independently conducted a comprehensive literature search across four databases, PubMed, EMBASE, Virtual Health Library (VHL), and medRxiv, covering all available records until October 29, 2024. To maximize sensitivity and specificity, we used the keywords “glaucoma,” “uveitis,” and “Rho-kinase inhibitors” in various combinations of searches. The search process was thoroughly documented in accordance with PRISMA guidelines, with medRxiv as a source of gray literature. Based on the research question and the selected databases, we conducted searches using controlled vocabulary terms such as “MeSH,” “Emtree,” and “DeCS,” adapting the strategies to meet the criteria of each database and scanning titles and abstracts. Full details of the search strategies are provided in Supplementary Material 2.

### Eligibility criteria for considering studies for this review

This systematic review included primary studies evaluating at least 10 eyes diagnosed with UG or OHT-SU. Eligible study designs encompassed case series, cohort studies, cross-sectional studies, and clinical trials. Articles written in English or Spanish—the languages spoken by all reviewers in our team—were eligible for full-text screening. Articles that were not available in full-text, studies performed in species other than humans, case reports, case series with fewer than 10 eyes, and analyses from secondary data sources (economic analyses, systematic and non-systematic reviews) were excluded from consideration.

### Criteria for participant inclusion and exclusion

For the systematic review, the inclusion criteria encompassed patients of any age, sex, or ethnicity with a confirmed diagnosis of UG or OHT-SU who were being treated with ROCK inhibitors as either naïve therapy or with a prior history of conventional hypotensive therapies. UG was defined as the presence of glaucomatous damage, either structural (such as increased cup-to-disc ratio or changes detected on Optical Coherence Tomography) or functional (visual field loss), in the context of IOP elevation [2]. In contrast, OHT-SU was defined as an IOP greater than 21 mmHg requiring treatment with at least one IOP-lowering medication without evidence of structural or functional glaucomatous damage [2]. Exclusion criteria included subjects diagnosed with other types of glaucoma that confound the outcomes of the review, as well as those lacking documented follow-up information on IOP during treatment.

### Primary and secondary outcomes

The primary objective of the study was to assess the mean reduction in IOP following treatment with ROCK inhibitors at various time points, both in controlled settings (efficacy) and non-controlled settings (effectiveness), comparing these values to baseline measurements and examining the frequency of ocular and systematic adverse effects (safety).

Secondary objectives included analyzing differences in the primary outcomes between real-world data and clinical trial results, comparing the efficacy among different ROCK inhibitors, identifying the additive effect of ROCK inhibitors in the medical management of patients with UG and OHT-SU, and evaluating the impact of ROCK inhibitor use on the need for glaucoma surgeries and their outcomes.

### Study selection

The search results were downloaded in Research Information Systems (RIS) format and imported into Zotero to create a comprehensive database of the retrieved articles. Duplicate records were initially filtered within Zotero. Subsequently, a second Microsoft Excel screening removed duplicates by cross-referencing author names, article titles, and DOIs. After duplicates were removed, the articles were assigned to 11 independent reviewers (BA-B, JC-L, AR-T, SDA-A, DSE-S, FA-G, AMC-R, VS-C, SG-G, JB-B, SG-V), all trained in the definitions employed, who were organized into five groups to conduct a structured peer review of the remaining articles. The screening process was conducted in two phases: first, titles and abstracts were reviewed to assess relevance, followed by a detailed evaluation of the full-text articles. Eligibility was determined based on predefined inclusion and exclusion criteria. Articles were categorized into “included,” “excluded,” or “in doubt” in a Microsoft Excel database. In cases of disagreement among the reviewers, all authors collectively re-evaluated the articles until consensus was reached. In cases where consensus remained elusive, uveitis specialists (ADLT) were consulted to reach a final decision.

### Data collection and risk of bias assessment

Articles selected for the study were organized and tracked using a Microsoft Excel spreadsheet. Each article was reviewed by 11 reviewers (BA-B, JC-L, AR-T, SDA-A, DSE-S, FA-G, AMC-R, VS-C, SG-G, JB-B, SG-V) to ensure relevance and alignment with the study objectives. Data extraction included key information such as the article title, author names, year of publication, DOI, study design, total number of patients and eyes with UG or OHT-SU, patient age, follow-up duration, type of ROCK inhibitor, anatomical classification, cause of uveitis [16], reported adverse effects (e.g., conjunctival hyperemia, transient hyphema, ocular pain, back rash, and blurred vision), and results on IOP control during treatment.

The risk of bias assessment was independently performed by two reviewers (BA-B, AR-T) using validated tools tailored to the methodological designs of the included studies (Supplementary Material 3). For the case series, the Hassan Murad criteria were used [17], evaluating four general elements: 1) population selection, 2) ascertainment of exposure or outcome, 3) causality, and 4) sufficient reporting detail. To simplify scoring, the following values are assigned: if the question was answered “yes,” we assigned a “low risk of bias”; if the question was answered “no,” we assigned a “high risk of bias,” and if the question was not applicable, we assigned “some concerns.” The study was considered with a “low risk of bias” if all items were scored with a “low risk” of bias, “some concerns” if any item was scored as “some concerns”, and “high risk of bias” if any item was scored as “high risk of bias.”

For cross-sectional studies, the modified Hoy et al. tool [18] was employed, comprising 10 items across four domains (selection, non-response, measurement, and analysis bias) and a summary risk of bias assessment. Items included: 1) target population of the study, 2) representation of the sampling frame, 3) sample selection, 4) probability of non-response, 5) source of data collection, 6) case definition, 7) parameter measurement, 8) consistency of data collection, 9) follow-up period, and 10) suitability of the numerator and denominator for the parameter of interest. Affirmative responses added one point. External validity was rated as “high” for scores from zero to one, “some concerns” for a score of two, and “low” for scores of three. Similarly, internal validity was rated as “high” for scores of zero to two, “some concerns” for a score of three, and “low” for scores of four. Studies were considered to be at “high risk of bias” if any domain (internal or external validity) was judged to be at “high risk of bias.”

For cohort studies, the Clinical Advances Through Research and Information Translation (CLARITY) tool was applied, assessing eight domains: 1) selection of exposed and unexposed cohorts, 2) assessment of exposure, 3) outcome of interest not present at baseline, 4) matching of exposed and unexposed, 5) prognostic factors, 6) assessment of outcome, 7) follow-up, and 8) co-interventions [19]. To simplify the appropriate design-related scoring of each item assessed in the CLARITY tool (“Definitely yes:” “Probably yes;” “Probably no;” “Definitely no”), we assigned the following values: if the question was answered with “Definitely yes,” it was assigned a “low” risk of bias; if the rating was “Probably yes” or “Probably no,” we used the term “some concerns”; if the rating was “Definitely not,” we assigned a “high risk” of bias. Studies were deemed to have a “high risk of bias” if any domain received a “high risk” rating.

The RoB-2 tool was used to assess the risk of bias in randomized controlled trials, assessing five specific bias domains: 1) randomization, 2) deviation from intended interventions, 3) missing data, 4) outcome measurement, and 5) selection of reported outcomes [20]. Figures were generated using the Robvis visualization tool 34 [21] (Supplementary Material 3).

### Data synthesis and analysis

A qualitative narrative synthesis of key findings on population characteristics was initially conducted by one author (BA-B) using validated tables that specified study designs, geographical locations, cohort sizes, demographic data, etiologies, medical treatments (e.g., PGAs, beta-blockers, α2-agonists, and carbonic anhydrase inhibitors), and surgical interventions. These tables were subsequently reviewed and refined by another author (GM-S) to ensure accuracy and completeness.

For the quantitative analysis, a meta-analysis of mean differences in IOP was conducted to compare baseline with measurements at different time points: one month, three months, twelve months, and the final follow-up. For the latter, the final follow-up duration (in months) was included as a moderator in the model.

Subgroup analyses were performed to evaluate variations in IOP reduction between real-world data and clinical trial findings and among different ROCK inhibitors. Additionally, the frequency of ocular and systemic adverse effects was analyzed using a proportion meta-analysis, with the numerator representing the number of reported adverse events and the denominator the total number of eyes treated with ROCK inhibitors.

Meta-analyses of mean differences were conducted at the patient level for IOP reductions. In contrast, proportion meta-analyses were performed at the eye level to estimate the prevalence of adverse effects, as these were the levels at which most studies reported outcomes. Studies lacking specific data required for meta-analysis were excluded from this process, but were still included in the systematic review. Only variables reported in at least two studies were eligible for inclusion in the meta-analysis. A 95% confidence interval (CI) was calculated for all analyses, and results were considered statistically significant at *p < *0.05.

Statistical heterogeneity among studies was assessed using the I^2^ statistic, with thresholds defined as low (I^2^ < 50%), moderate (I^2^ = 50–75%), and high (I^2^ > 75%). Given the methodological differences between controlled trials and observational studies—including distinct entry criteria and patient selection processes—the type of study was prioritized over the degree of heterogeneity when selecting the analytical model [22] (Supplementary Material 4). Consequently, a common-effects model was applied to data consisting exclusively of controlled trials, whereas a random-effects model was used for observational data. All statistical analyses were performed using the R package dmetar (version 0.0.9000) (dmetar).

### Ethical considerations

This study adheres to the ethical principles for research involving human subjects as established in the Declaration of Helsinki, the Belmont Report, and Colombian Resolution 008430 of 1993. Due to the nature of the study, which involved analyzing previously published data, formal approval from an ethics committee was not required.

## Results

### Study selection

**Fig. 1:**
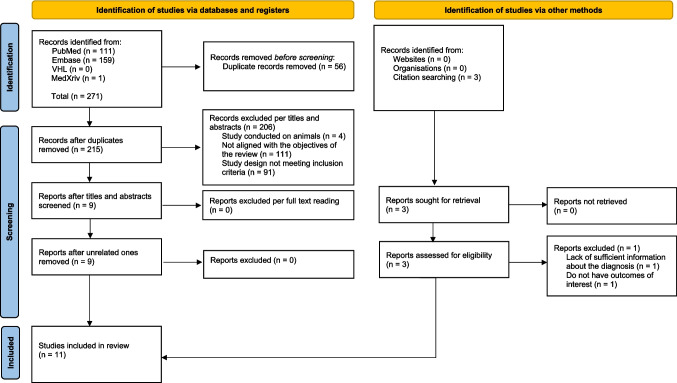
PRISMA flow diagram

Initially, 271 articles were identified through the database search. Of these, 56 duplicates were removed. After screening titles and abstracts, 206 articles were excluded for failing to meet the inclusion criteria. An additional search using citation tracking identified three more articles, one of which was excluded for not reporting outcomes of interest. Finally, 11 articles were deemed eligible for inclusion in the systematic review (Fig. 1). The number of studies included in the meta-analyses varied depending on the specific variables analyzed.

### Study and population characteristics

Among the 11 included studies, two were cross-sectional, and both were assessed as having a “low risk” of bias. Three studies were case series, all categorized as having a “high risk” of bias. Four cohort studies were included, each evaluated as having “some concerns.” Additionally, two randomized controlled trials were included, categorized as having a “high risk” of bias (Supplementary Material 3). Geographically, most studies were conducted in Asia, specifically in Japan (eight studies) and India (one study). The remaining two studies were conducted in the United States. Population sizes varied from 8 to and 109 subjects.

A total of 383 participants and 256 eyes were included across the 11 studies analyzed. It is important to note that while some studies reported outcomes per eye, others reported data exclusively per patient and did not specify the number of affected eyes. Therefore, the total number of eyes reflects only those explicitly reported in the included studies and does not necessarily match the number of participants. Among the 196 patients from the seven studies reporting sex (representing 51.2% of the total participants), 99 were male (50.5%, 99/196), and 97 were female (49.5%, 97/196). Furthermore, pooling the means and standard deviations (SDs) from the 10 studies reporting age, the mean age of the population was 64.24 ± 15.16 years. Additionally, 347 (90.6%) participants were categorized as having UG, while 36 (9.4%) were identified as having OHT-SU.

Regarding the etiology of uveitis, 83 eyes (32.4%) lacked a reported etiology. Out of the 173 cases reporting etiology, 87 (50.3%) were categorized as non-infectious, 71 eyes (41.4%) as idiopathic, and 15 (8.7%) as infectious. Specific non-infectious etiologies included sarcoidosis within 34 eyes (39.1%, 34/87), Vogt-Koyanagi-Harada disease (12/87 eyes, 13.7%), and Behçet’s disease (8/87 eyes, 9.2%). The most frequent infectious etiologies included herpes simplex virus infection (12/15 eyes, 80%) and cytomegalovirus infection (3/15 eyes, 20%) (Table 1), indicating the heterogeneous uveitic etiologies that can produce UG or OHT-SU.

Regarding medical treatment, 8 (3.1%) eyes were on monotherapy with conventional hypotensive agents, 23 (8.9%) were receiving dual therapy, 11 (4.3%) were on triple therapy, and 7 (2.7%) were on quadruple therapy, reflecting the variable use of different hypotensive regimens to achieve adequate pressure control in these patients. Additionally, before ROCK inhibitors, 8 eyes (3.1%) underwent surgical intervention, including trabeculectomy. Moreover, 5 eyes (1.9%) required surgical intervention during ROCK inhibitor administration, including glaucoma surgery, underscoring the risk of surgical necessity in this patient population (Table 1).

**Table 1:** Study and population characteristics

Author	Study design	Risk of bias	Geographical location	Cohort size (Pt/eyes)	Age of UG and OHT-SU presentation (Mean ± SD)	Sex (M/F)	Etiology, n (%)	Medicaltreatments(eyes)	Surgical interventions(eyes)
Oydanich, 2024 10.1016/j.jcjo.2023.05.009	Cross-sectional	Low	North-America -USA	14/18	64.5± 21.2	ND	ND	ND	ND
Oydanich 202410.1080/09273948.2022.2145313	Case-series	High	North-America -USA	13/17	60.1±16.6	5/8	ND	DT: 10	ND
Tanihara, 202210.1007/s12325-021-02023-y	Cohort	Someconcerns	Asia - Japan	56/NID	69.1± 12.7	ND	ND	ND	ND
Mimura, 202210.2174/18743641-V16-E2206201	RandomizedClinical-trial	High	Asia - Japan	8/8	69.9 ± 9.4	5/3	Idiopathic: 2 (25)Non-infectious: 4(50)Infectious: 2 (25)	TT: 3QT: 5	Trabeculectomy beforetreatment: 8
Nagpal,202110.7860/JCDR/2021/48878.14851	RandomizedClinical-trial	High	Asia - India	20/ND	56.2± 16.3	13/7	ND	ND	ND
Tanihara, 201910.1007/s12325-018-0863-1	Cohort	Someconcerns	Asia - Japan	55/48	69.1± 12.7	ND	ND	ND	ND
Kusuhara, 201810.1007/s00417-018-3933-9	Case-series	High	Asia - Japan	19/21	N/A	10/9	Idiopathic: 7 (33.3)Non-infectious: 12(51.1)Infectious: 2 (9.5)	NM: 3MT: 3DT: 9TT:4QT:2	ND
Yasuda, 201710.1371/journal.pone.0185305	Cross-sectional	Low	Asia - Japan	16/20	62.1±13.2	5/11	Idiopathic: 9 (45)Non-infectious: 7 (35)Infectious: 4 (20)	ND	ND
Futakuchi, 202010.1038/s41598-020-66928-4	Cohort	Somconcerns	Asia - Japan	ND/109	63 ± 14	56/53	Idiopathic: 50 (45.9)Non-infectious: 54(49.5)Infectious: 5 (4.6)	ND	ND
Yanai, 202310.18240/ijo.2023.06.11	Case-series	High	Asia - Japan	11/15	53.6± 16.5	5/6	Idiopathic: 3 (20)Non-infectious: 10(66.7)Infectious: 2 (13.3)	NM: 5MT: 2DT: 4TT: 4	Glaucoma surgery aftertreatment: 5
Tanihara, 201910.1186/s12886-020-01490-1	Cohort	Someconcerns	Asia - Japan	62/ND	69.1±12.7	ND	ND	ND	ND

USA: United States of America, Pt: Patients, ND: No data reported, UG: Uveitic glaucoma, OHT-SU: Ocular hypertension secondary to uveitis, SD: Standard deviation, M: Male, F: Female, NM: No medication, MT: Mono Therapy, DT: Dual therapy, TT: Triple therapy, QT: Quadruple therapy

### Impact of Rho-Kinase inhibitors on intraocular pressure reduction

**Fig. 2:**
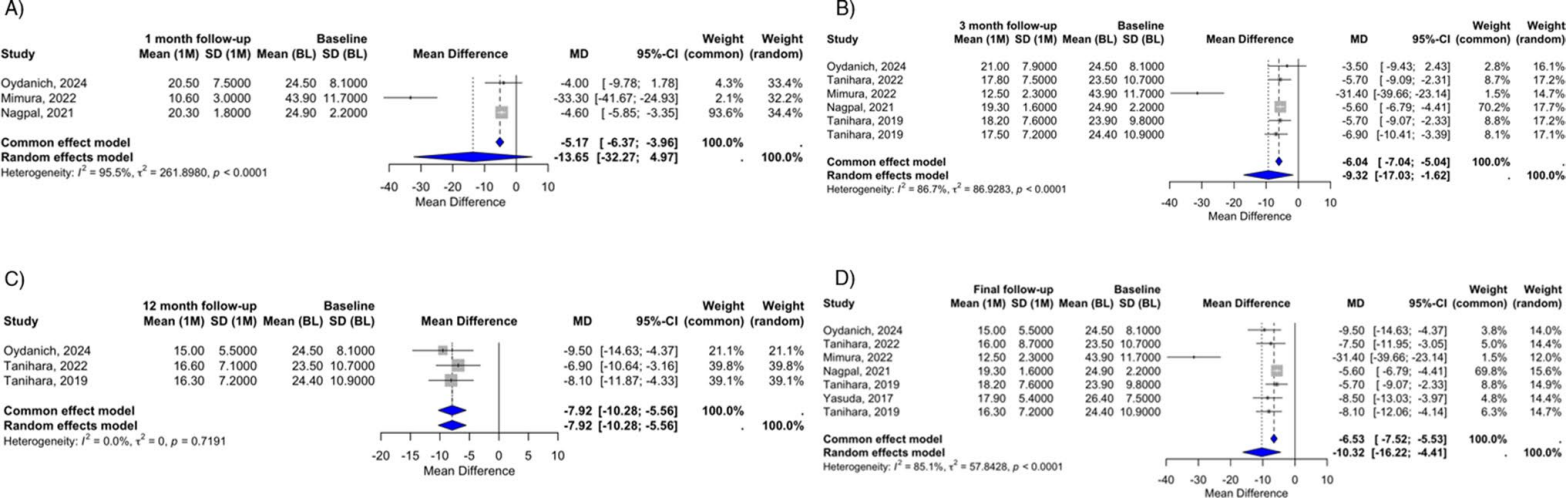
Mean IOP reduction with ROCK inhibitors across different follow-up periods in UG and OHT-SU. (**A**). 1 month follow-up, (**B**). 3 month follow-up, (**C**). 12 month follow-up, (**D**). Final follow-up

We first sought to evaluate the impact of ROCK inhibitors in reducing IOP at different follow-up points. At the 1-month follow-up, across three studies, the pooled mean IOP reduction was −13.65 mmHg, but it was not statistically significant (95% CI: −32.27 to 4.97). Substantial heterogeneity was observed (I^2^ = 95.5%, *p < *0.0001), reflecting considerable variability in individual results ranging from −4.00 to −33.30 mmHg. High heterogeneity limited the certainty of the pooled estimate at this early follow-up point (Fig. 2A).

At the 3-month follow-up, the pooled mean difference in IOP was −9.32 mmHg (95% CI: −17.03 to −1.62) within seven studies, achieving statistical significance. However, heterogeneity remained high (I^2^ = 86.7%, *p < *0.0001), indicating that variability in study designs and populations influenced the results, which ranged from −3.50 to −31.40 mmHg (Fig. 2B). At the 12-month follow-up, the analysis revealed a statistically significant pooled mean difference of −7.92 mmHg (95% CI: −10.28 to −5.56) within three studies. Heterogeneity was very low (I^2^ = 0%, *p* = 0.71), reflecting a high level of consistency in the effect estimates at this time point (Fig. 2C). At the final follow-up, which included seven studies with a mean follow-up duration of 16.43 ± 11.83 months, a significant reduction of IOP was documented at −10.32 mmHg (95% CI: −4.41 to −16.22). Heterogeneity returned high (I^2^ = 85.1%, *p < *0.0001) (Fig. 2D).

### Variability in treatment effects: controlled trials vs real-world data

**Fig. 3:**
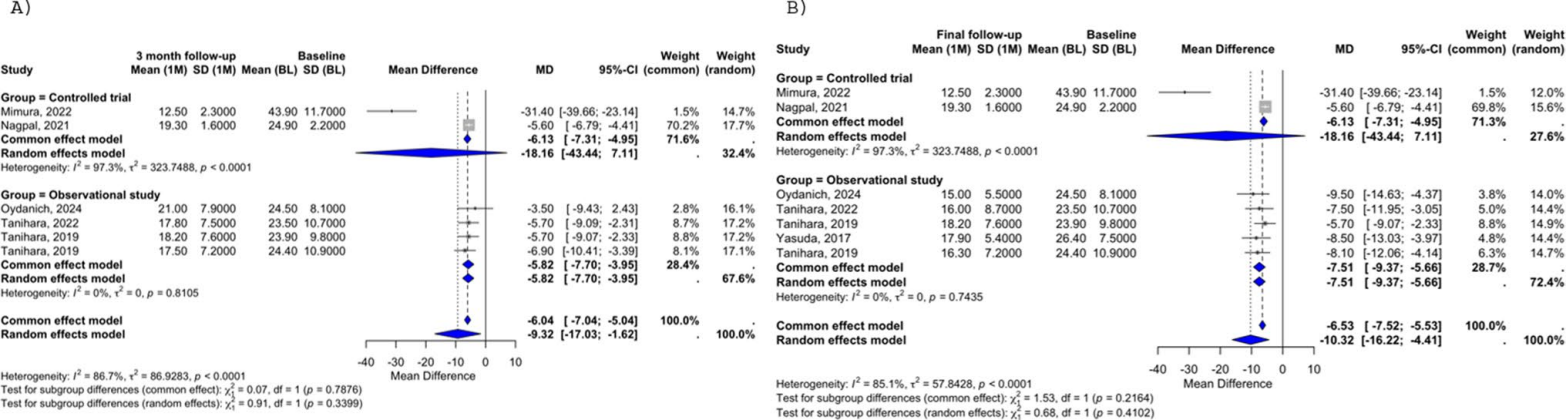
IOP reduction in controlled trials and observational studies. (**A**). 3- month follow-up, (**B**). Final follow-up

We evaluated the mean difference in IOP reduction between controlled and real-world scenarios by comparing the results of controlled trials and observational studies at follow-up points at which at least two studies were available for each group. At the 3-month follow-up with six studies, controlled trials demonstrated a significant mean reduction in IOP of −6.13 mmHg (95% CI: −7.31 to −4.95) when analyzed using the common-effect model. However, under the random-effects model, the reduction was not significant (−18.16 mmHg; 95% CI: −43.44 to 7.11); I^2^ = 97.3%, *p < *0.0001 (Fig. 3A). For observational studies reflecting real-world clinical practice, the pooled mean difference was significant under both common and random effect models at −5.82 mmHg (95% CI: −7.70 to −3.95), I^2^ = 0%, p = 0.8105 (Fig. 3A). These results underscore the significant IOP-lowering effect of ROCK inhibitors in practical settings, helping optimize IOP control in these challenging patient populations with a pooled effect (considering both scenarios: clinical trials and observational studies) of −6.04 mmHg (95% CI: −7.04 to—5.04) in the common-effect model and −9.32 mmHg (95% CI: −17.03 to −1.62) in the random-effects model.

Similar trends were observed at the final follow-up, based on seven studies with a mean follow-up duration of 16.43 ± 11.83 months. Controlled trials demonstrated a significant mean reduction in IOP of −6.13 mmHg (95% CI: −7.31 to −4.95) under the common-effect model. However, this reduction was not statistically significant when analyzed using the random-effects model (−18.16 mmHg; 95% CI: −43.44 to 7.11); I^2^ = 0%, *p* = 0.8105); I^2^ = 97.3%, *p < *0.0001. For observational studies, IOP reductions were significant under both models, with a pooled mean difference of −7.51 mmHg (95% CI: −9.37 to −5.66); I^2^ = 0%, *p* = 0.74), underscoring that the use of these novel drugs impacts clinical practice, significantly reducing the IOP. When data from both controlled trials and observational studies were combined, the IOP-lowering effect remained significant, with reductions of −6.53 mmHg (95% CI: −7.52 to—5.33) in the common-effect model and −10.32 mmHg (95% CI: −16.22 to −4.41) in the random-effects model (Fig. 3B).

When comparing the populations of controlled trials and observational studies, notable differences were evident. Controlled trials often included patients with stricter eligibility criteria, such as uncontrolled IOP despite maximum therapy [23], while observational studies encompassed more heterogeneous populations with prior glaucoma interventions [24–26]. These differences in baseline characteristics may partly explain the observed heterogeneity in the pooled results, with I^2^ = 86.7%, *p < *0.0001 at three-month follow-up and I^2^ = 85.1%, *p < *0.0001 at final follow-up (Fig. 3B).

**Fig. 4:**
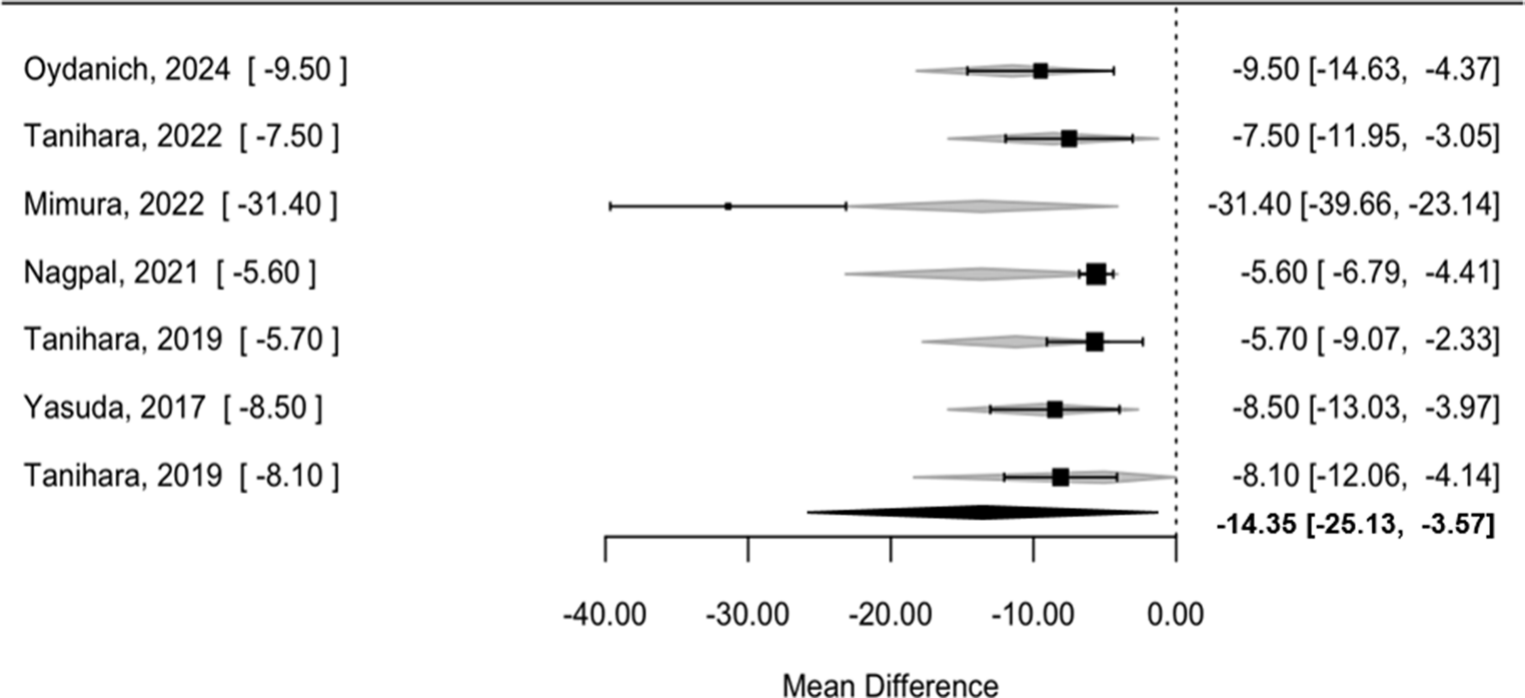
Forest plot of IOP reduction with ROCK inhibitors with type of study as moderator

Since follow-up duration was consistent across studies (3 months), heterogeneity can be attributed more to differences in population characteristics than time. Consequently, the type of study (clinical trial vs observational) was incorporated as a moderator in the analysis. This adjustment revealed a significant reduction in IOP of −14.35 mmHg (95% CI: −25.13 to −3.57; I^2^ = 93.9%, p = 0.009) (Fig. 4). emphasizing the impact of baseline variability on treatment outcomes regardless of the type of study.

### Safety of ROCK inhibitors

We conducted a meta-analysis using a proportional method to assess the frequency of adverse events across the four studies that reported them. The most frequently observed adverse event was conjunctival hyperemia, with a pooled prevalence of 7% (95% CI: 3%−17%). Other reported events include ocular pain (4%, 95% CI: 1%−13%), hyphema (5%, 95% CI: 1%−15%), blurred vision (3%, 95% CI: 1%−11%), tearing (3%, 95% CI: 1%−11% and back rash (4%, 95% CI: 1%−12%) The low prevalence of this adverse events highlights the safety of the use of ROCK inhibitors in UG and OHT-SU. No significant heterogeneity was detected between studies for any adverse event (I^2^ = 0%), reflecting a high level of consistency (Table 2).

**Table 2:** Frequency of ocular and systemic adverse effects after RHO inhibitors use in patients with uveitic glaucoma or ocular hypertension secondary to uveitis

Adverse Event	Number of Studies	Number of eyes	Pooled prevalence 95% CI	p-value	I^2^	Selected model
Conjunctival hyperemia	5	3	6% (3% - 15%)	0.79	0%	Random effects model
Ocular pain	5	1	4% (1% - 11%)	0.97	0%	Random effects model
Hyphema	5	1	4% (1% - 13%)	0.79	0%	Random effects model
Blurred vision	5	0	3% (1% - 10%)	0.99	0%	Random effects model
Tearing	5	0	3% (1% - 10%)	0.99	0%	Random effects model
Black rash	5	1	1% (4% - 11%)	0.99	0%	Random effects model

### Differences in ROCK inhibitors: ripasudil and netarsudil

The efficacy and safety profiles of ripasudil and netarsudil have been studied individually. While no studies to date have directly compared these agents, evidence from individual investigations offers valuable insights into their potential effectiveness and safety.

Ripasudil has demonstrated significant IOP-lowering efficacy. Kusuhara et al. reported an average IOP reduction of 3 mmHg at 1 month of treatment and up to 2 mmHg at 1 year, with sustained effects over time. These effects were accompanied by a low discontinuation rate, with mild adverse events, such as conjunctival hyperemia [14]. Additionally, Yanai et al. reported a significant reduction in IOP, from 26.4 ± 2.9 mmHg to 13.7 ± 3.3 mmHg at 3 months, which remained stable over a two-year follow-up period (*p < *0.0001) [27]. In a randomized trial, Nagpal et al. reported a 22.7% reduction in IOP at 12 weeks, with no significant adverse events (*p* = 0.001) [28].

Similarly, netarsudil has shown promising IOP-lowering efficacy in UG patients. Odyanich et al. reported that over 50% of treated eyes achieved IOP reductions greater than 20%. Although conjunctival hyperemia was more commonly reported with netarsudil, it did not appear to compromise patient adherence to treatment [15]. In a retrospective cohort study, an average reduction of 9.5 mmHg (36%) from baseline after 12 months of treatment (*p* = 0.01) [28].

### Additive effect of ROCK inhibitors used in the medical therapy of patients with UG and OHT-SU

We aimed to explore whether ROCK inhibitors provide an additional IOP-lowering effect when combined with other glaucoma medications compared to their use alone. While a meta-analysis could not be conducted due to limited data, the available evidence identified in this systematic review suggests that these agents enhance the IOP-lowering effect of conventional treatments.

Yanai et al. reported that 66.7% of eyes treated with ripasudil were already on glaucoma medications before initiating therapy. The addition of ripasudil led to a significant decrease in IOP, from 26.4 ± 2.9 mmHg to 13.7 ± 3.3 mmHg after three months of treatment [27]. Similarly, Mimura et al. found that ripasudil reduced dependence on multiple medications in some patients. Notably, after trabeculectomy, the mean number of antiglaucoma medications was significantly lower in the ripasudil group (0.1 ± 0.3) compared to the control group (1.6 ± 1.5) at three months of follow-up (*p* = 0.015), emphasizing its potential to reduce reliance on conventional hypotensive eyedrops [23]. Likewise, Odyanich et al. documented a similar trend in a cohort of 17 eyes with UG, where the average number of antiglaucoma medications required during follow-up decreased [15].

## Discussion

This study evaluated the efficacy, effectiveness, and safety of ROCK inhibitors in the management of UG and OHT-SU. UG and OHT-SU continue to pose significant therapeutic challenges, given their inflammatory nature, variable response to conventional treatments, and the risk of surgical failure. In recent years, ROCK inhibitors have attracted growing interest due to their distinct mechanism of action and potential anti-inflammatory properties [11, 12, 29]. Although several studies have reported their efficacy in lowering IOP, their role in the specific context of OHT-SU had not been systematically assessed.

Our findings highlight the potential of these drugs as a promising therapeutic option. A significant IOP reduction was observed at three months and was sustained through 12 months, highlighting its sustained effect over time. These outcomes, along with the low incidence of adverse events, suggest a favorable safety and effectiveness profile. Moreover, combining ROCK inhibitors with standard therapies demonstrated an additive effect and a favorable safety profile. However, since their initial effect may take time to develop, they could be considered mainly in patients who do not achieve their IOP goals with conventional treatment.

### Efficacy

The unique mechanism of action of ROCK inhibitors, which directly targets the TM [12], may explain their ability to sustainably reduce IOP in patients with UG and OHT-SU.

In previous clinical trials, including those by Mimura et al. and Nagpal et al., a consistent and long-lasting reduction IOP has been demonstrated [23, 30]. However, a key difference is that the initial effect observed in some studies with ripasudil may be less pronounced during the first month. Although the pharmacokinetics of these drugs are not yet fully understood, this difference in initial response may be attributable to their specific mechanisms of action or interindividual variability [11].

Our results reinforce this observation, showing that efficacy is consolidated at three months, with an additive effect when combined with other standard therapies. Furthermore, studies suggest that ROCK inhibitors offer significant efficacy under controlled conditions, mainly when used as adjunctive therapy in patients who fail to achieve IOP goals with conventional treatments. Their real impact, however, will depend on a better understanding of their mechanisms of action across patient subgroups on their integration into broader therapeutic regimens.

### Effectiveness

Real-world studies have highlighted the effectiveness of ROCK inhibitors in reducing IOP. Research by Odyanich et al. demonstrated that netarsudil treatment led to a decrease in IOP of more than 20% in more than half of the treated eyes, with this effect persisting for a 1 year [15]. Likewise, Yanai et al. observed that in patients already receiving glaucoma therapy, adding ripasudil significantly lowered IOP by 12.7 mmHg (48.1%) within three months [27].

Our findings align with these reports, confirming that the benefits demonstrated in controlled trials translate into significant effectiveness in clinical practice. The pooled mean IOP reduction in real-world settings was −9.32 mmHg (95% CI: −17.02 to −1.62) at three months and −7.92 (95% CI: −10.28 to −5.56) mmHg at 12 months, suggesting that ROCK inhibitors retain their effectiveness beyond experimental settings and remain a viable option for routine glaucoma management.

### Differences between clinical trials and real-world results

Clinical trials provide a rigorous and controlled evaluation of the efficacy of ROCK inhibitors in lowering IOP in patients with UG and OHT-SU. However, the results obtained in these studies do not always reflect the real impact of these drugs in daily clinical practice. This discrepancy between the efficacy observed in clinical trials and the effectiveness in the real world can be attributed to multiple factors.

The differences between controlled trials and observational studies highlight the tension between internal validity and external applicability. Controlled trials, through strict eligibility criteria and standardized protocols, provide robust evidence of efficacy, but may not fully reflect the variability of real-world clinical settings, where comorbidities, inconsistent adherence, and combined treatments are common. Notably, some of the available controlled trials in this evidence base included patients who had previously failed maximal medical therapy. This reflects the characteristics of the published literature rather than a methodological limitation, and contributes to greater heterogeneity than typically expected in randomized designs. In contrast, in observational studies, patients often receive ROCK inhibitors alongside other glaucoma therapies in flexible regimens, which may influence the magnitude and consistency of IOP reduction. This methodological variability likely contributes to differences in outcomes and underscores the importance of interpreting pooled estimates within the context of study design, entry criteria, and population characteristics.

Our results reflect this difference, as the average IOP reduction observed in the real world was consistent with previous studies but with a broader range of responses. This underscores the need to understand better the factors influencing the clinical effectiveness of ROCK inhibitors and to optimize their integration into individualized treatment strategies.

### Safety

ROCK inhibitors exhibited a favorable safety profile, with adverse events primarily limited to the ocular surface. The most frequently reported side effects we re conjunctival hyperemia, (6%; 95% CI: 3%−15%), followed by ocular discomfort and blurred vision. These effects were mild, localized, and generally well tolerated, differentiating ROCK inhibitors from other classes of IOP-lowering drugs that may be associated with systemic adverse effects or a higher incidence of ocular surface changes [31].

Compared to conventional therapies such as PGAs, beta-blockers, α2-agonists, and carbonic anhydrase inhibitors, ROCK inhibitors exhibit a distinct safety profile. For example, prostaglandin analogs are known to cause conjunctival hyperemia and periorbital changes [32–34]. Carbonic anhydrase inhibitors are associated with cases of color vision changes, bilateral transient myopia, and choroidal detachment [35, 36]. In contrast, ROCK inhibitors have demonstrated limited systemic absorption an adverse impact mainly localized to the ocular surface.

Blepharitis has been reported as a common adverse effect associated with ripasudil use. In the literature studies evaluating ripasudil in various types of secondary glaucoma have described blepharitis outside the uveitic glaucoma setting [26]. Real-world surveillance studies, have identified blepharitis as one of the most frequent adverse events during ripasudil treatment, with its incidence increasing with longer exposure and representing a common reason for treatment discontinuation [25, 26]. In addition, observational studies including heterogeneous secondary glaucoma populations have reported blepharitis in specific subtypes, such as exfoliation glaucoma and steroid-induced glaucoma [37]. Although blepharitis was not consistently reported *among* patients with uveitic glaucoma in the studies included in our review, this may be attributable to the relatively short follow-up periods in these studies, which generally did not exceed 12 months, rather than to a true absence of this adverse event. Given that blepharitis has been consistently observed with longer-term ripasudil use in other glaucoma populations, it is plausible that this adverse effect may also occur in patients with uveitic glaucoma under prolonged ripasudil exposure; however, this could not be adequately evaluated within the timeframe of the current evidence base.

Our findings are consistent with previous reports, indicating that although ROCK inhibitors may induce conjunctival hyperemia more frequently than some conventional treatments, the severity and clinical impact of this effect are usually mild [38–41]. Furthermore, although exact discontinuation rates were not consistently reported, the available evidence does not indicate substantial differences in tolerability between ROCK inhibitors and other commonly used therapies.

### Limitations

One of the main limitations is the limited number of available studies evaluating these drugs, particularly in patients with UG and OHT-SU. Most of the data come from studies with small sample sizes or designs that do not always allow extrapolation of the results to broader populations. This highlights the need for additional studies with greater statistical power and long-term follow-up.

In addition, the studies analyzed exhibit substantial heterogeneity in patient populations, treatment regimens, duration of follow-up, and criteria for evaluating therapeutic response. These variations can influence the interpretation of the results and limit the possibility of establishing universally applicable clinical recommendations.

Another relevant aspect is the global availability of ROCK inhibitors. These drugs have only been approved in countries such as Japan and the United States. They do not, however, share the same treatment options [42–45]. This restricts their access in many regions, limiting their implementation in clinical practice outside these territories. Expanding their approval and availability in other countries will be crucial to evaluating their impact on different populations and treatment settings. Additionally, the high cost of ROCK inhibitors has been a major barrier to their widespread use. Expanding regulatory approval and improving cost-effectiveness will be essential for broader implementation and for assessing their impact across diverse healthcare systems.

### Future studies

While ROCK inhibitors have demonstrated a positive impact on IOP reduction in UG and OHT-SU, multiple research areas could contribute to a better understanding of their role in treating these conditions.

One of the main lines of future research would be to compare the different types of ROCK inhibitors available directly. Currently, ripasudil and netarsudil are the most studied, but systematic direct comparisons have not been performed to assess whether there are significant differences in efficacy, safety, or long-term adherence. This approach would allow the specific characteristics of each drug to be identified and their clinical use to be optimized.

Another key area is the evaluation of the efficacy and effectiveness of ROCK inhibitors in glaucoma or secondary ocular hypertension of different etiologies. It is well known that treatment response can vary depending on the underlying disease mechanism. For example, in ocular hypertension secondary to viral infections, the increase in IOP is often related to inflammatory trabeculitis [46–48]. However, in other types of secondary glaucoma, such as corticosteroid-induced glaucoma, the mechanism may differ substantially, potentially influencing the therapeutic response. Studies analyzing these differences would help better define which scenarios ROCK inhibitors could be most effective.

Finally, long-term studies are required to evaluate their impact on the progression of glaucomatous damage and their potential neuroprotective role, given that some mechanisms of action of ROCK inhibitors could have beneficial effects beyond simply reducing IOP.

## Conclusion

ROCK inhibitors represent a promising therapeutic strategy for UG and OHT-SU, demonstrating significant IOP-lowering efficacy and a favorable safety profile. Their additive effect with conventional treatments suggests a role as adjunctive therapy in patients with uncontrolled IOP. However, the heterogeneity of current studies and the limited global availability highlight the need for further high-quality trials to optimize clinical use and broaden their accessibility. Future research should focus on comparative efficacy, long-term safety, and real-world effectiveness to establish their definitive role in managing glaucoma associated with uveitis.

## Supplementary Information

Below is the link to the electronic supplementary material.

Supplementary file1 (DOCX 29 KB)

Supplementary file2 (DOCX 19 KB)

Supplementary file3 (DOCX 127196 KB)

Supplementary file4 (DOCX 18 KB)

## Data Availability

This study is based on secondary analysis of previously published data. All information and results can be reproduced using the search strategy provided in Supplementary Material 2 and the meta-analysis workflow detailed in Supplementary Material 4.
