# Supplementary material for: Efficacy, effectiveness, and safety of rho-kinase inhibitors in uveitic glaucoma and ocular hypertension secondary to uveitis: a systematic review and meta-analysis
Source: Graefes Arch Clin Exp Ophthalmol. 2026 Feb 3;264(6):1527–40. doi: 10.1007/s00417-025-07111-1 (PMC13197265; doi:10.1007/s00417-025-07111-1)
Supplement: Supplementary file 2 — Supplementary file2 (DOCX 19 KB) [file 417_2025_7111_MOESM2_ESM.docx]

**Supplementary Material 2. Search strategy.**

Search Strategy PUBMED – DATE 29 October 2024

| TERMS | SEARCH |
| --- | --- |
| Rho Kinase inhibitors  30,806 | (("Rho kinase inhibitors"[MeSH Terms] OR "ROCK inhibitors"[MeSH Terms] OR "Rho-associated protein kinase"[MeSH Terms] OR "Rho kinase"[Title/Abstract] OR "ROCK"[Title/Abstract] OR Ripasudil[Title/Abstract] OR Netarsudil[Title/Abstract] OR "K-115"[Title/Abstract] OR "AR-13503"[Title/Abstract]) |
| Uveitic glaucoma  103,366 | ("glaucoma"[MeSH Terms] OR "uveitis"[MeSH Terms] OR "ocular hypertension"[MeSH Terms] OR "secondary glaucoma"[Title/Abstract] OR "uveitic glaucoma"[Title/Abstract] OR "ocular hypertension secondary to uveitis"[Title/Abstract] OR "glaucoma associated with uveitis"[Title/Abstract] OR "ocular hypertension"[Title/Abstract] OR "eye pressure"[Title/Abstract] OR "ocular inflammation"[Title/Abstract])) |
| Efficacy or effectiveness  1,924,828 | ("intraocular pressure"[MeSH Terms] OR "IOP"[Title/Abstract] OR "ocular pressure"[Title/Abstract] OR "pressure reduction"[Title/Abstract] OR "pressure control"[Title/Abstract] OR efficacy[Title/Abstract] OR effectiveness[Title/Abstract] OR "treatment outcome"[Title/Abstract] OR "clinical outcome"[Title/Abstract] OR "real-world effectiveness"[Title/Abstract])) |
| Safety  1,143,011 | ("safety"[Title/Abstract] OR "adverse effects"[MeSH Terms] OR "side effects"[Title/Abstract] OR "tolerability"[Title/Abstract] OR "ocular side effects"[Title/Abstract] OR "systemic side effects"[Title/Abstract] OR "conjunctival hyperemia"[Title/Abstract] OR "ocular irritation"[Title/Abstract] OR "corneal endothelial damage"[Title/Abstract] OR "visual disturbance"[Title/Abstract] OR "adverse reaction"[Title/Abstract] OR "adverse event"[Title/Abstract] OR "blurred vision"[Title/Abstract] OR "itching"[Title/Abstract] OR "increased tearing"[Title/Abstract] OR "rash"[Title/Abstract] OR "subconjunctival hemorrhage"[Title/Abstract]) |
| Search strategy  Results:  111 | (("Rho kinase inhibitors"[MeSH Terms] OR "ROCK inhibitors"[MeSH Terms] OR "Rho-associated protein kinase"[MeSH Terms] OR "Rho kinase"[Title/Abstract] OR "ROCK"[Title/Abstract] OR Ripasudil[Title/Abstract] OR Netarsudil[Title/Abstract] OR "K-115"[Title/Abstract] OR "AR-13503"[Title/Abstract])  AND ("glaucoma"[MeSH Terms] OR "uveitis"[MeSH Terms] OR "ocular hypertension"[MeSH Terms] OR "secondary glaucoma"[Title/Abstract] OR "uveitic glaucoma"[Title/Abstract] OR "ocular hypertension secondary to uveitis"[Title/Abstract] OR "glaucoma associated with uveitis"[Title/Abstract] OR "ocular hypertension"[Title/Abstract] OR "eye pressure"[Title/Abstract] OR "ocular inflammation"[Title/Abstract]))  AND ("intraocular pressure"[MeSH Terms] OR "IOP"[Title/Abstract] OR "ocular pressure"[Title/Abstract] OR "pressure reduction"[Title/Abstract] OR "pressure control"[Title/Abstract] OR efficacy[Title/Abstract] OR effectiveness[Title/Abstract] OR "treatment outcome"[Title/Abstract] OR "clinical outcome"[Title/Abstract] OR "real-world effectiveness"[Title/Abstract]))  AND ("safety"[Title/Abstract] OR "adverse effects"[MeSH Terms] OR "side effects"[Title/Abstract] OR "tolerability"[Title/Abstract] OR "ocular side effects"[Title/Abstract] OR "systemic side effects"[Title/Abstract] OR "conjunctival hyperemia"[Title/Abstract] OR "ocular irritation"[Title/Abstract] OR "corneal endothelial damage"[Title/Abstract] OR "visual disturbance"[Title/Abstract] OR "adverse reaction"[Title/Abstract] OR "adverse event"[Title/Abstract] OR "blurred vision"[Title/Abstract] OR "itching"[Title/Abstract] OR "increased tearing"[Title/Abstract] OR "rash"[Title/Abstract] OR "subconjunctival hemorrhage"[Title/Abstract]) |

Search Strategy EMBASE – DATE 29 October 2024

| TERMS | SEARCH |
| --- | --- |
| Rho Kinase inhibitors  103,879 | ('rho kinase inhibitor'/exp OR 'rho associated protein kinase inhibitor' OR 'rock inhibitor' OR 'rho kinase' OR 'rock' OR ripasudil OR netarsudil OR 'k-115' OR 'ar-13503') |
| Uveitic glaucoma  22,304 | ('uveitic glaucoma'/exp OR 'secondary glaucoma'/exp OR 'ocular hypertension'/exp OR 'glaucoma associated with uveitis' OR 'ocular hypertension secondary to uveitis' OR 'uveitic glaucoma' OR 'ocular inflammation' OR 'eye pressure') |
| Efficacy or effectiveness  4,517,645 | ('intraocular pressure'/exp OR 'ocular pressure' OR iop OR 'pressure reduction' OR 'pressure control' OR efficacy OR effectiveness OR 'treatment outcome' OR 'clinical outcome' OR 'real-world effectiveness') |
| Safety  3,040,430 | ('safety'/exp OR 'adverse effect'/exp OR 'side effect' OR tolerability OR 'ocular side effect' OR 'systemic side effect' OR 'conjunctival hyperemia'/exp OR 'ocular irritation' OR 'corneal endothelial damage' OR 'visual disturbance' OR 'adverse reaction' OR 'adverse event' OR 'blurred vision' OR itching OR 'increased tearing' OR rash OR 'subconjunctival hemorrhage') |
| Search strategy  Results:  159 | ('rho kinase inhibitor'/exp OR 'rho associated protein kinase inhibitor' OR 'rock inhibitor' OR 'rho kinase' OR 'rock' OR ripasudil OR netarsudil OR 'k-115' OR 'ar-13503') AND ('uveitic glaucoma'/exp OR 'secondary glaucoma'/exp OR 'ocular hypertension'/exp OR 'glaucoma associated with uveitis' OR 'ocular hypertension secondary to uveitis' OR 'uveitic glaucoma' OR 'ocular inflammation' OR 'eye pressure') AND ('intraocular pressure'/exp OR 'ocular pressure' OR iop OR 'pressure reduction' OR 'pressure control' OR efficacy OR effectiveness OR 'treatment outcome' OR 'clinical outcome' OR 'real-world effectiveness') AND ('safety'/exp OR 'adverse effect'/exp OR 'side effect' OR tolerability OR 'ocular side effect' OR 'systemic side effect' OR 'conjunctival hyperemia'/exp OR 'ocular irritation' OR 'corneal endothelial damage' OR 'visual disturbance' OR 'adverse reaction' OR 'adverse event' OR 'blurred vision' OR itching OR 'increased tearing' OR rash OR 'subconjunctival hemorrhage') |

Search Strategy VHL– DATE 29 October 2024

| TERMS | SEARCH |
| --- | --- |
| Rho Kinase inhibitors  0 | (("Rho kinase inhibitors" OR "inhibidores de Rho quinasa" OR "ROCK inhibitors" OR "Rho-associated protein kinase" OR "kinasa de Rho" OR Ripasudil OR Netarsudil OR "K-115" OR "AR-13503") |
| Uveitic glaucoma  0 | ("uveitic glaucoma" OR "glaucoma uveítico" OR "secondary glaucoma" OR "glaucoma secundario" OR "ocular hypertension" OR "hipertensión ocular" OR "glaucoma asociado a uveítis" OR "hipertensión ocular secundaria a uveítis" OR uveitis OR "inflamación ocular" OR "presión intraocular")) |
| Efficacy or effectiveness  0 | ("intraocular pressure" OR "presión intraocular" OR IOP OR "ocular pressure" OR "reducción de presión" OR "control de presión" OR eficacia OR effectiveness OR efectividad OR "treatment outcome" OR "resultados del tratamiento" OR "clinical outcome" OR "real-world effectiveness" OR "efectividad en el mundo real")) |
| Safety  0 | ("safety" OR seguridad OR "adverse effects" OR "efectos adversos" OR "side effects" OR "efectos secundarios" OR tolerabilidad OR tolerability OR "ocular side effects" OR "efectos secundarios oculares" OR "systemic side effects" OR "efectos secundarios sistémicos" OR "conjunctival hyperemia" OR hiperemia OR "ocular irritation" OR "irritación ocular" OR "corneal endothelial damage" OR "daño endotelial corneal" OR "visual disturbance" OR "trastorno visual" OR "adverse reaction" OR "reacción adversa" OR "adverse event" OR "evento adverso" OR "blurred vision" OR "visión borrosa" OR itching OR prurito OR "increased tearing" OR "aumento del lagrimeo" OR rash OR "erupción cutánea" OR "subconjunctival hemorrhage" OR "hemorragia subconjuntival")) |
| Search strategy  Results:  0 | (("Rho kinase inhibitors" OR "inhibidores de Rho quinasa" OR "ROCK inhibitors" OR "Rho-associated protein kinase" OR "kinasa de Rho" OR Ripasudil OR Netarsudil OR "K-115" OR "AR-13503")  AND ("uveitic glaucoma" OR "glaucoma uveítico" OR "secondary glaucoma" OR "glaucoma secundario" OR "ocular hypertension" OR "hipertensión ocular" OR "glaucoma asociado a uveítis" OR "hipertensión ocular secundaria a uveítis" OR uveitis OR "inflamación ocular" OR "presión intraocular"))  AND ("intraocular pressure" OR "presión intraocular" OR IOP OR "ocular pressure" OR "reducción de presión" OR "control de presión" OR eficacia OR effectiveness OR efectividad OR "treatment outcome" OR "resultados del tratamiento" OR "clinical outcome" OR "real-world effectiveness" OR "efectividad en el mundo real"))  AND ("safety" OR seguridad OR "adverse effects" OR "efectos adversos" OR "side effects" OR "efectos secundarios" OR tolerabilidad OR tolerability OR "ocular side effects" OR "efectos secundarios oculares" OR "systemic side effects" OR "efectos secundarios sistémicos" OR "conjunctival hyperemia" OR hiperemia OR "ocular irritation" OR "irritación ocular" OR "corneal endothelial damage" OR "daño endotelial corneal" OR "visual disturbance" OR "trastorno visual" OR "adverse reaction" OR "reacción adversa" OR "adverse event" OR "evento adverso" OR "blurred vision" OR "visión borrosa" OR itching OR prurito OR "increased tearing" OR "aumento del lagrimeo" OR rash OR "erupción cutánea" OR "subconjunctival hemorrhage" OR "hemorragia subconjuntival")) |

Search Strategy MedXRiv– DATE 29 October 2024

| TERMS | SEARCH |
| --- | --- |
| Rho Kinase inhibitors  11,455 | Rho kinase inhibitors OR ROCK inhibitors |
| Uveitic glaucoma  56,632 | uveitic glaucoma OR secondary glaucoma OR glaucoma associated with uveitis |
| Search strategy  Results:  1 | Rho kinase inhibitors OR ROCK inhibitors AND uveitic glaucoma OR secondary glaucoma OR glaucoma associated with uveitis |
