# Supplementary material for: Efficacy, effectiveness, and safety of rho-kinase inhibitors in uveitic glaucoma and ocular hypertension secondary to uveitis: a systematic review and meta-analysis
Source: Graefes Arch Clin Exp Ophthalmol. 2026 Feb 3;264(6):1527–40. doi: 10.1007/s00417-025-07111-1 (PMC13197265; doi:10.1007/s00417-025-07111-1)
Supplement: Supplementary file 4 — Supplementary file4 (DOCX 18 KB) [file 417_2025_7111_MOESM4_ESM.docx]

**Supplementary Material 4: Sensitivity analysis**

**Heterogeneity measures definitions**

**I2:** It indicates the percentage of variation across studies due to heterogeneity rather than chance and quantifies inconsistency among the results of different studies ^1,2^.

- 0: No heterogeneity (perfect consistency among studies).
- 0-50: Low heterogeneity.
- 51-75%: Moderate heterogeneity.
- 76% or higher: High heterogeneity.

**τ² (Tau-squared):** It reflects the variance between study effects in a meta-analysis. In contrast to I², which provides a percentage, τ² gives an estimate of the data's scale. Low τ² values indicate a slight variance between the study effect sizes, suggesting that the true effect is consistent across studies. Conversely, high τ² values indicate substantial variance between study effect sizes, suggesting that the true effect varies significantly across studies. While there are no strict thresholds for what constitutes "low" or "high" τ², general guidelines suggest ^1,2^.

- < 0.04: low heterogeneity.
- 0.04 to 0.36: Moderate heterogeneity.
- > 0.36: High heterogeneity.

These values can vary depending on the context and field of study. For instance, even a τ² value of 0.04 might be considered moderate in clinical research if the studies are highly precise and consistent in their methodologies.

**OR (Odds Ratio):** This is a measure of association used in meta-analyses to compare the odds of an outcome occurring in one group to the odds of it occurring in another group. It synthesizes the results of multiple studies that have assessed the same association ^1,2^.

- OR=1: No difference in odds between the groups.
- OR>1: The outcome is more likely in the experimental group.
- OR<1: The outcome is less likely in the experimental group.

**Common effect model metanalysis:** A common effect model (also known as a fixed effect model) assumes a true effect size shared among all the included studies. It is used when it is reasonable to assume that all the studies estimate the same underlying effect ^1,2^.

**Random effect model metanalysis:** A random effects model assumes that the true effect size varies among studies. This model accounts for variability within and between studies; therefore, it is recommended when heterogeneity is high ^1,2^.

**References**

1. Viechtbauer W. Conducting Meta-Analyses in R with the metafor Package. J Stat Soft 2010;36. Available at: http://www.jstatsoft.org/v36/i03/ [Accessed July 13, 2024].

2. Jonathan J D, Julian PT H, Douglas G A. Cochrane Handbook for Systematic Reviews of Interventions: Chapter 10: Analysing data and undertaking meta-analyses. 2008th ed. Cochrane Book Series. John Wiley & Sons Available at: Cochrane Book Series. John Wiley & Sons.
