# Supplementary material for: Efficacy, effectiveness, and safety of rho-kinase inhibitors in uveitic glaucoma and ocular hypertension secondary to uveitis: a systematic review and meta-analysis
Source: Graefes Arch Clin Exp Ophthalmol. 2026 Feb 3;264(6):1527–40. doi: 10.1007/s00417-025-07111-1 (PMC13197265; doi:10.1007/s00417-025-07111-1)
Supplement: Supplementary file 3 — Supplementary file3 (DOCX 127196 KB) [file 417_2025_7111_MOESM3_ESM.docx]

**Supplementary Material 3**

**Supplementary Figure 1. Case series risk of bias.**

**Supplementary Figure 2. Cross-sectional risk of bias.**

**Supplementary Figure 3. Cohort studies risk of bias.**

**Supplementary Figure 4. Randomized clinical trial risk of bias.**
